# Overcoming the strength–formability trade-off in high strength steels via cryogenic treatment

**DOI:** 10.1038/s41598-022-19521-w

**Published:** 2022-09-14

**Authors:** Gyeongbae Park, A. Zargaran, J. K. Oh, T. T. T. Trang, N. J. Kim

**Affiliations:** 1 Korea Institute of Materials Science, Changwon, Republic of Korea; 2 Graduate Institute of Ferrous & Energy Materials Technology, Pohang University of Science and Technology, Pohang, Republic of Korea; 3 POSCO Technical Lab., Gwangyang, Republic of Korea

**Keywords:** Mechanical properties, Metals and alloys

## Abstract

High strength steels are becoming more important than ever before for automotive applications to reduce the weight of automobiles and to ensure the safety of passengers. Since increased strength usually results in degraded formability, however, cold forming of high strength steels into final shapes remains a challenge to both automotive manufacturers and suppliers. Here we report novel alloy and processing design concepts that can impart high strength to cold-formable steels, which deviates from the traditional approach of improving the formability of high strength steels. Such designed steel subjected to a designed processing route shows an excellent combination of formability and strength as well as crashworthiness, which is crucial for the safety of passengers in the automobiles. The alloy and processing design concepts used in the present study are based on the utilization of thermally induced austenite to martensite transformation, which imparts high strength to cold-formable austenite by cryogenic treatment.

## Introduction

Advanced high strength steels (AHSS) play an important role in automobile manufacturing since they can meet increasingly stringent requirements of automobile industries for the safety and lightweighting of vehicles^1,2^. Although there are several variants of light materials such as Al and Mg alloys that can be used for structural components in automobiles, the characteristics of AHSS such as their high strength and low manufacturing cost make them materials of choice over other materials^3–5^. Among several grades of AHSS, hot press forming (HPF) steels, also called press hardening steels, have received a great deal of attention since they offer superior tensile properties along with good dimensional accuracy due to their unique processing route consisting of high temperature forming within austenite region followed by cooling in a die to form low temperature hard phases such as martensite or bainite^6–8^. However, such a processing route has a poor production efficiency and moreover these HPF steels generally suffer from liquid metal embrittlement (LME) or microcracking due to an interaction between coating and substrate during high temperature forming^9–14^. Therefore, a new type of high strength steels is needed, which can be formed to a final shape at low temperatures and does not suffer from LME or microcracking associated with high temperature forming.

Fulfilling the requirement for a combination of high strength and high formability is a challenging task since conventional approaches that improve formability have adverse effects on strength or vice versa. In this regard, the use of metastable austenite as a major constituent phase would be a viable approach since austenite, being a face-centered cubic (fcc) phase, can provide good formability at room temperature and also can provide high strength when the transformation of austenite to martensite is enforced by cooling below their martensite start (*M*_*s*_) temperature^15–19^ after forming. Stabilization of austenite at room temperature has traditionally been achieved by adding a fairly large amount of austenite stabilizing elements such as in the cases of classical transformation-induced plasticity (TRIP) steels^20^ and high Mn steels^15,16,21^, which are usually subjected to austenitization followed by water quenching (WQ). Considering that the stability of austenite in hypoeutectoid steels would be increased when annealed in (ferrite + austenite) intercritical region as compared to austenitization treatment, a fairly large amount of austenite can be retained in steels with leaner alloy compositions after intercritical annealing (IA) followed by water quenching, which is the basic alloy design concept for medium Mn TRIP steels^22–33^. These medium Mn TRIP steels usually contain 30–70 vol% austenite depending on the alloy compositions and show good formability; however, their yield strength is much lower than that of HPF steels. The yield strength of medium Mn steels, as mentioned previously, can be significantly increased when austenite to martensite transformation is promoted by cooling to low temperature, i.e., by cryogenic treatment. Here, the critical issue is that while austenite to martensite transformation should occur by cryogenic treatment to achieve high strength, a substantial amount of austenite should also be retained after cryogenic treatment to ensure crashworthiness of the final components.

In the present study, by utilizing cryogenic treatment, an attempt has been made to develop the steels that show good formability in the intercritically annealed (IAed) condition as well as high strength in the cryogenic treated condition through the transformation of retained austenite to martensite. The model alloy containing 10Mn, 2Al, and 0.2C was fabricated and its microstructure and mechanical properties were evaluated after IA of hot rolled sheets with various annealing conditions as well as after immersion in liquid N_2_.

## Experimental procedure

### Fabrication and thermomechanical treatment process

30 kg ingot of the steel with a nominal composition of Fe–10Mn–2Al–0.2C (analyzed composition of Fe–9.9Mn–2.0Al–0.2C in wt%) was fabricated using vacuum induction melting. After homogenization at 1150 °C for 2 h, it was hot rolled between 1050 and 850 °C followed by water quenching to produce 3 mm thick sheet with a total reduction of 95%. The hot-rolled sheet was intercritically annealed at 715 °C, 725 °C, and 735 °C for 30 min followed by water quenching. In addition, the IAed sheets were subjected to immersion into liquid N_2_ (cryogenic treatment) for 1 h to see its effect on microstructure and tensile properties. Cryogenic treated sheets were also heat treated at 170 °C for 20 min and air cooled to simulate the paint baking cycle of automotive components^34–36^.

### Microstructure analyses

Microstructure of the steel was analyzed by X-ray diffraction (XRD), electron backscatter diffraction (EBSD), and transmission electron microscopy (TEM). EBSD analyses were conducted at the middle of rolled sheets (perpendicular to transverse direction) using a field emission SEM (FE-SEM, JEOL JSM-7100F). EBSD measurements were performed at an accelerating voltage of 15 kV and a step size of 0.05–0.2 µm. EBSD data were then analysed using OIM analysis software ver.8.0 (EDAX Inc.). High angle boundaries (15°–62°) are highlighted in the inverse pole figure (IPF) and phase maps. XRD scans were performed on a Bruker D8 Advance diffractometer using Cu K_α_ radiation. The obtained XRD data were used to calculate the volume fraction of each constituent phase following the direct comparison method suggested by De et al.^37^. EBSD was also used to measure the volume fraction of the blocky austenite so that the volume fraction of the lath austenite can be indirectly calculated by subtracting the volume fraction of the blocky austenite measured by EBSD from the total volume fraction of austenite measured by XRD. TEM thin foils were prepared by twin-jet polishing (Tenupol-5, Struers) in a 95% acetic acid + 5% perchloric acid solution at 40 V. TEM examinations were carried out on a JEOL JEM-2100F field emission electron microscope at an operating voltage of 200 kV. The compositions of the phases were measured by energy dispersive X-ray spectroscopy (EDS) in STEM mode.

### Tensile and bending tests

Tensile specimens were cut along the rolling direction with a gauge length, width, and thickness of 25 mm, 6 mm, and 2.5 mm, respectively, according to ASTM standard E8/E8M. Tensile test was conducted at an initial strain rate of 10^–3^ s^−1^, using an Instron 8801 universal testing machine.

Bending tests were performed following the guidelines of the ISO 7438^38^ of the International Organization for Standardization (ISO) and VDA 238-100^39^ of the German Association of the Automotive Industry. The rectangular specimens with a length of 60 mm, a width of 30 mm, and a thickness of 1.5 mm were subjected to three-point bending, where the bending axis is parallel with the rolling direction of the sheet, at a punch speed of 20 mm min^−1^ with a punch radius of 0.4 mm and a roller diameter of 30 mm. Bending angle was measured by the protractor. The *R/t* ratio was measured as the smallest ratio of the punch radius (*R*) to the sheet thickness (*t*) that results in a crack-free bend during bending to an angle of 90°. The punch radii used were 3 mm to 8 mm in 0.5 mm intervals.

## Results and discussion

### Microstructural evolution

**Figure 1:**
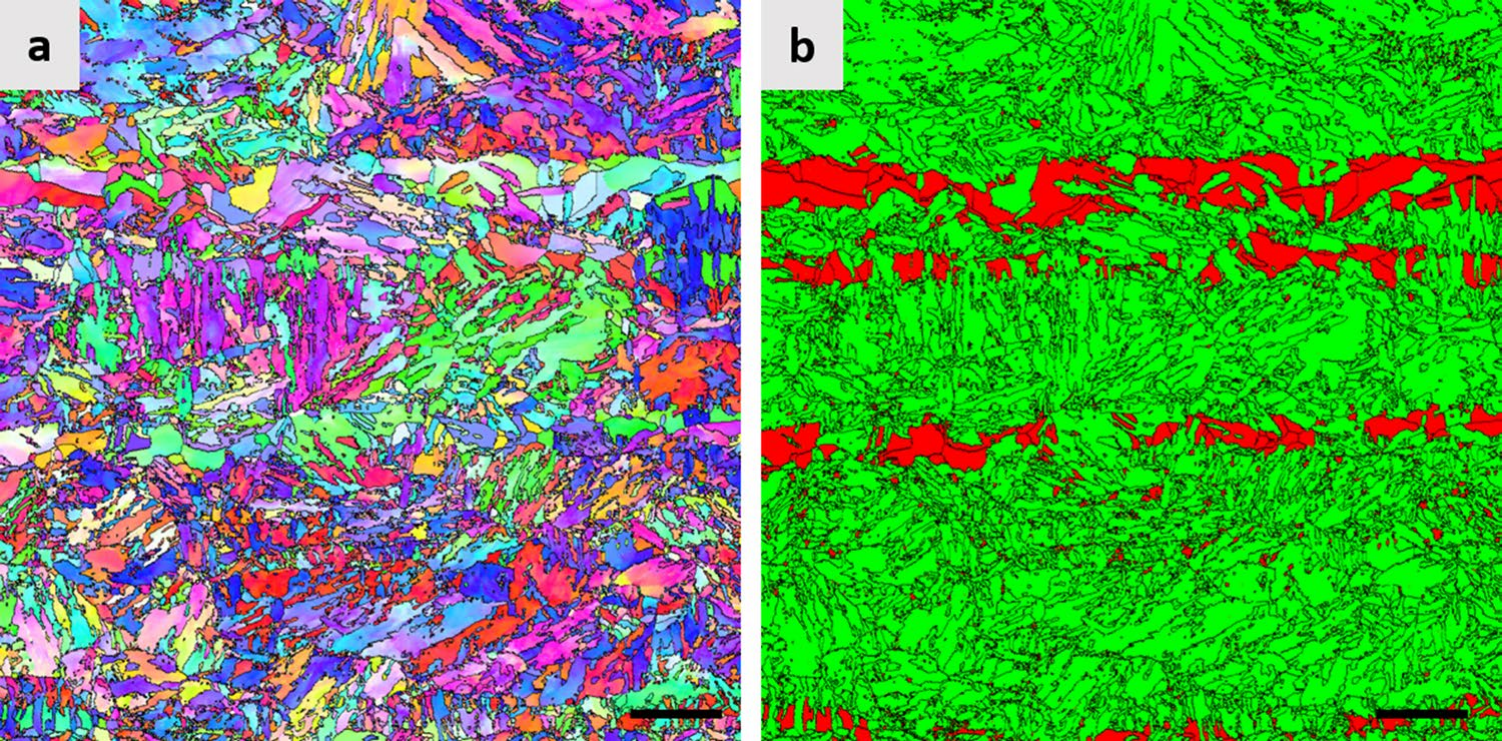
Microstructure of the steel in as-hot rolled condition. (**a**) EBSD IPF map and (**b**) EBSD phase map. In (**b**), austenite and bcc phases are marked by red and green colors, respectively. Scale bar of images corresponds to 10 μm.

Typical microstructure of the steel in as-hot rolled condition is shown in Fig. 1. As shown by EBSD phase map (Fig. 1b), the microstructure consists mostly of body-centered cubic (bcc) phase (marked by green color) with some austenite (marked by red color). It can be seen that most of bcc phase is martensite having the morphology of lath martensite. One interesting feature of the microstructure is that most of austenite in as-hot rolled condition is present in the form of band, which is due to a segregation of Mn frequently observed in medium Mn steels^25^.

**Figure 2:**
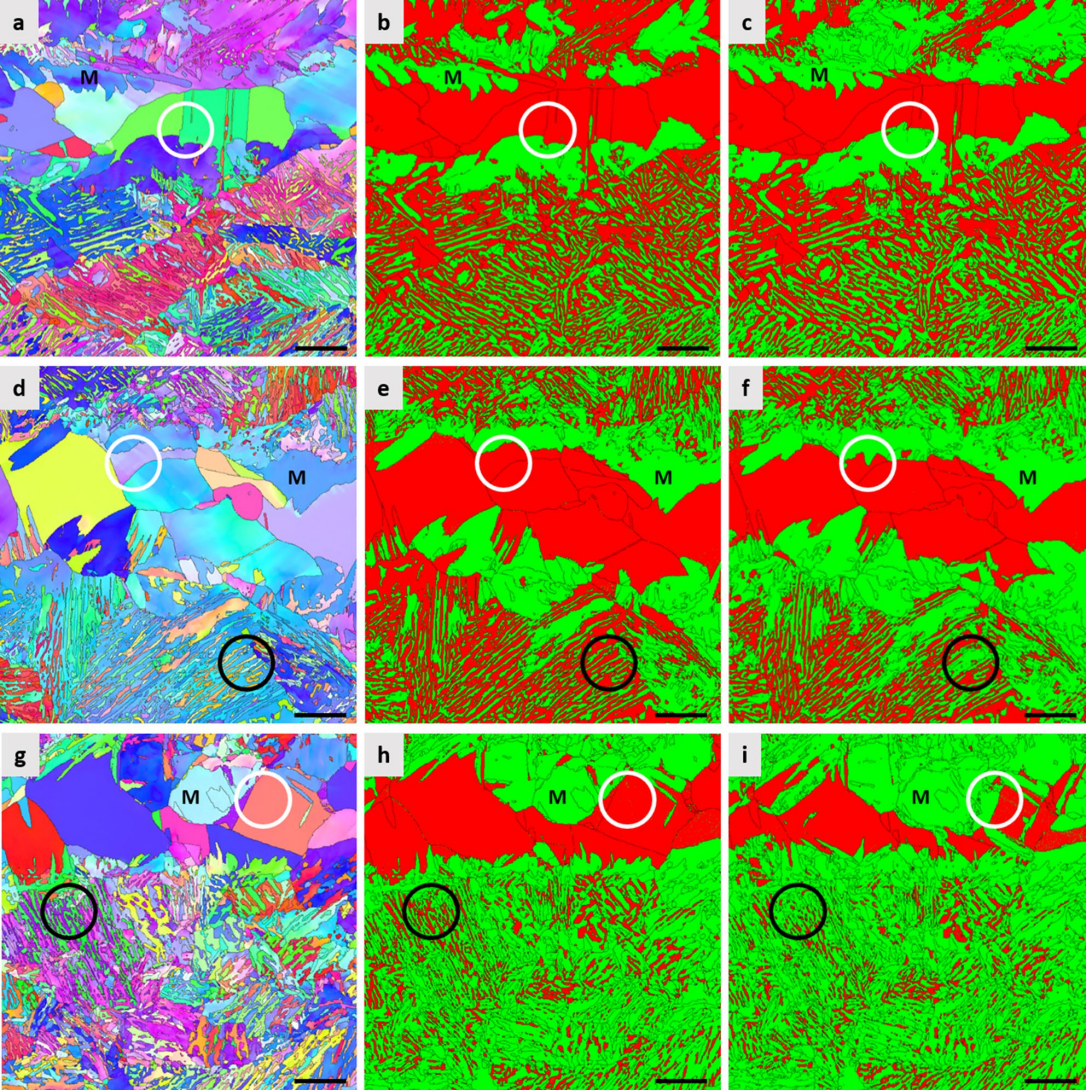
Microstructure of the specimens intercritically annealed (IAed) at different temperatures (**a–c**). The specimen IAed at 715 °C; EBSD IPF map (**a**) and EBSD phase map (**b**) after water quenching (WQ), and EBSD phase map after cryogenic treatment (**c**). (**d–f**) The specimen IAed at 725 °C; EBSD IPF map (**d**) and EBSD phase map (**e**) after WQ, and EBSD phase map after cryogenic treatment (**f**). (**g–i**) The specimen IAed at 735 °C; EBSD IPF map (**g**) and EBSD phase map (**h**) after WQ, and EBSD phase map after cryogenic treatment (**i**). Austenite and bcc phases are marked by red and green colors, respectively. Scale bar of images corresponds to 5 μm.

Figure 2 shows the microstructure of the specimens subjected to IA at temperatures of 715 °C (referred to as the ‘IA715-WQ’) (Fig. 2a,b), 725 °C (referred to as the ‘IA725-WQ’) (Fig. 2d,e), and 735 °C (referred to as the ‘IA735-WQ’) (Fig. 2g,h) followed by WQ. The microstructure of the IAed specimens resembles the as-hot rolled microstructure such that there is a presence of blocky austenite in band form. Besides the blocky austenite, there is also a presence of austenite having the lath morphology within bcc matrix, which resulted from the precipitation of austenite along lath boundaries of the prior martensite in as-hot rolled specimen^26^. It can also be seen that there are featureless bcc grains in the vicinity of the blocky austenite (e.g., marked by M in Fig. 2). Measurement of the fraction of austenite in each heat treatment condition by XRD (Table 1) shows that there is only a slight increase in the fraction of austenite when IA temperature increases from 715 °C (61%) to 725 °C (63%), suggesting that the IA temperature giving the largest fraction of austenite would be near 725 °C based on thermodynamic modeling^40^. A further increase in IA temperature (to 735 °C) results in a rather large decrease in the fraction of austenite (from 63 to 47%). Such variation in the fraction of austenite with IA temperature of the present steel shows the trend same as the one frequently observed in other IAed medium Mn steels^24,27,28^, due to a change in austenite stability resulting from varying degrees of solute partitioning. The respective fractions of the blocky and lath austenite were calculated following the procedure described in the Methods section and are listed in Table 1, showing that the majority of austenite is present in the lath morphology.

**Table 1:** Volume fraction of retained austenite in the specimens intercritically annealed (IAed) at different temperatures subjected to water quenching (WQ) and cryogenic treatment (LN_2_).

Specimen	IA715			IA725			IA735		
	WQ	LN_2_	TR (%)	WQ	LN_2_	TR (%)	WQ	LN_2_	TR (%)
Total γ fraction (%)	61	56	8	63	49	22	47	27	43
Blocky γ fraction (%)	14	10	29	18	13	28	12	6	50
Lath γ fraction (%)	47	46	2	45	36	20	35	21	40
γ fraction after fracture (%)	14	15	–	15	13	–	16	12	–

*TR* transformation ratio.

**Figure 3:**
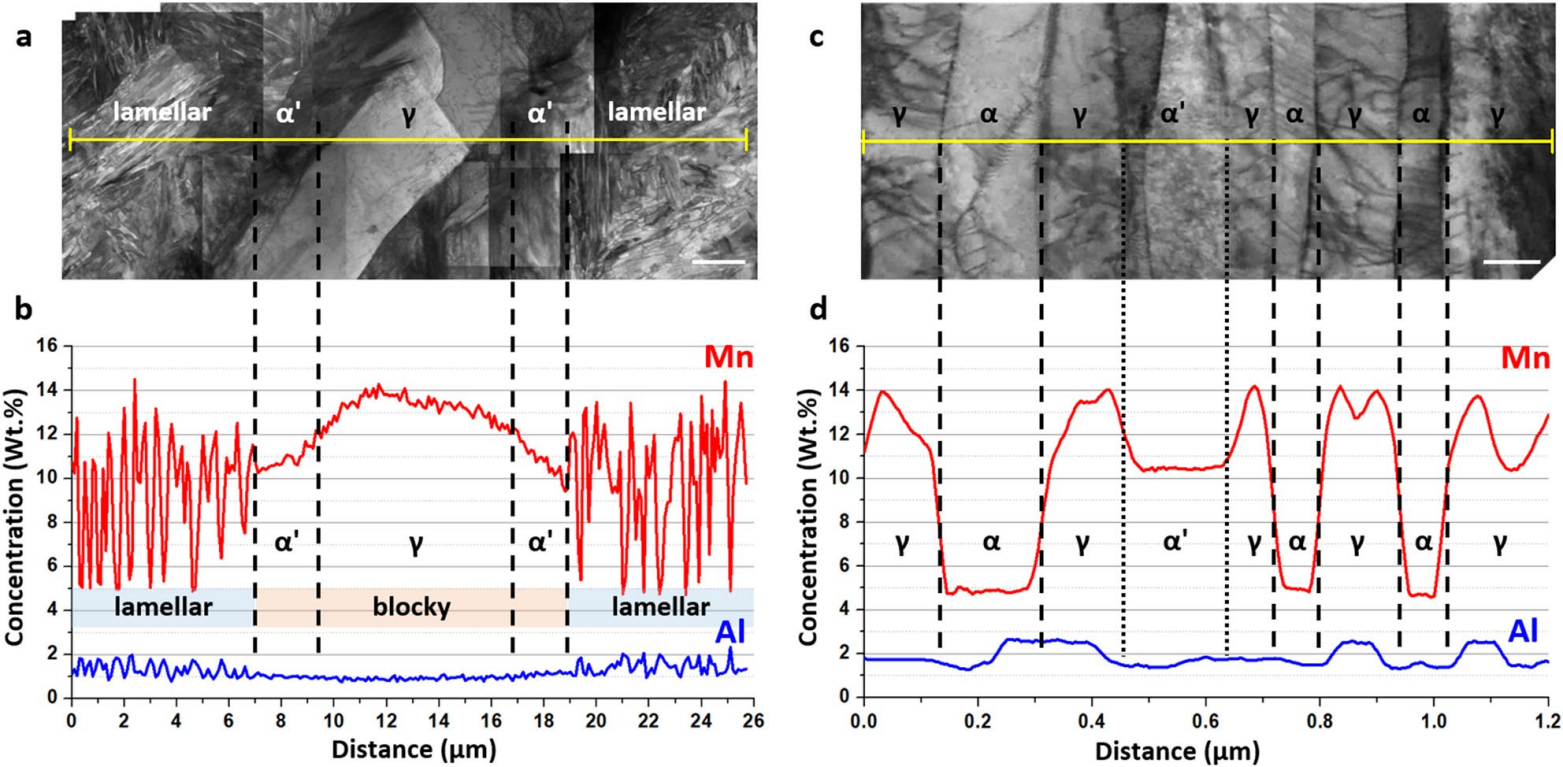
TEM micrographs showing the morphologies and compositions of the blocky austenite, lath austenite, and their surrounding areas in the specimen intercritically annealed at 715 °C followed by water quenching. (**a**) Morphology of the blocky austenite and its surrounding area. (**b**) Mn and Al concentration profiles of the blocky austenite and its surrounding area. (**c**) Morphology of the lath austenite and its surrounding area. (**d**) Mn and Al concentration profiles of the lath austenite and its surrounding area. Scale bars in (**a**) and (**c**) correspond to 2 µm and 100 nm, respectively.

Representative TEM micrographs of blocky austenite and its surrounding area are shown in Fig. 3. EDS analysis (Fig. 3b) shows that the blocky austenite is enriched with Mn (~ 13.1 wt%), but depleted in Al (~ 0.9 wt%). In the vicinity of the blocky austenite, there exist martensite islands having the Mn content (~ 10.8 wt.%) lower than that in the blocky austenite. Comparison with EBSD maps (Fig. 2a,b) shows that the featureless bcc grains (e.g., marked by M in Fig. 2a,b) surrounding the blocky austenite are indeed martensite. Further away, there is a lamellar structure consisting of ferrite, martensite, and austenite laths, whose details are shown in Fig. 3c. Similar to the blocky austenite, lath austenite is also enriched with Mn, whose Mn content is similar (~ 12.8 wt%) to that of the blocky austenite. Nearby the lath austenite, both ferrite and martensite laths are present, whose identification was made by comparing their dislocation densities (Fig. 3c). Mn content of the lath martensite is ~ 10.4 wt%, which is lower than that of the lath austenite, but higher than that (~ 6.0 wt%) of the nearby lath ferrite. The presence of martensite in the IA715-WQ specimen was not expected since thermodynamic calculation suggests that below the IA temperature forming the largest fraction of retained austenite, there would be no transformation of austenite to martensite upon quenching to room temperature^28,40^. It is believed that the formation of martensite in the present case is due to an inhomogeneous distribution of Mn in the microstructure, typical of hot-rolled medium Mn steels^25^. Analyses of the IA725-WQ and IA735-WQ specimens show that their microstructure is basically identical to that of the IA715-WQ specimen, except that Mn content in both blocky and lath austenite decreases with an increase in IA temperature as expected.

After cryogenic treatment, there is a decrease in the amount of austenite for all IA conditions. As shown in Table 1, the largest change in the fraction of austenite upon cryogenic treatment, i.e., transformation ratio, is found in the IA735 specimen, followed by the IA725 and IA715 specimens. To clearly visualize the change in the microstructure upon cryogenic treatment, the areas observed after IA-WQ were also subjected to microstructural analyses after cryogenic treatment, which are shown in Fig. 2c (for the IA715-LN_2_), Fig. 2f (for the IA725-LN_2_), and Fig. 2i (for the IA735-LN_2_). It can be seen that both types of austenite, coarse blocky austenite (for example, marked by white circles) and fine lath austenite (for example, marked by black circles) except in the case of the IA715 specimen (Fig. 2b,c), transform to martensite when subjected to cryogenic treatment. It is noted that not the whole grain of the blocky austenite transforms to martensite upon cryogenic treatment; instead only a portion of austenite grain, particularly located near the periphery of the prior austenite, transforms to martensite. This is mainly because of a concentration gradient in the blocky austenite; there is less amount of Mn near the periphery than the center of the blocky austenite, making the former less stable than the latter. Thus, it can be thought that the blocky austenite retained after cryogenic treatment would be quite stable and beneficial for the mechanical properties of cryogenic treated specimens.

As shown in Table 1, interestingly, quantitative analyses show that the degree of change in the fraction of austenite by cryogenic treatment is different depending on the type of austenite, which is also dependent on the IA temperature. In general, the lath austenite appears to be more thermally stable than the blocky austenite, although their relative stability changes with the IA temperature such that while the lath austenite shows a much lower transformation ratio (i.e., higher thermal stability) than the blocky austenite in the IA715 specimen, they show more or less similar transformation ratios in the IA735 specimen. It is generally known that the stability of austenite depends on several factors: its size^41^ and morphology^42^, and solute contents^43,44^. Although the blocky austenite is more enriched with Mn than the lath austenite, it is thought that the coarse blocky morphology of the blocky austenite is responsible for its lower thermal stability than that of the lath austenite, which is consistent with the result of previous studies showing that the lath austenite is more stable than the blocky austenite^22,23,45^. Nevertheless, as mentioned previously, the thermal stability of the lath austenite becomes more similar to that of the blocky austenite as the specimens are subjected to higher IA temperatures, indicating that factors other than size and morphology would also play important roles in determining the thermal stability of austenite in the IA725 and IA735 specimens. It is well known that the austenite should have a critical amount of solutes to be retained^43,44^. Considering that the lath austenite has a steep concentration gradient, only a narrow portion of the lath austenite would be retained upon cryogenic treatment, which becomes more evident in the specimens subjected to higher IA temperature. On the other hand, the blocky austenite has a shallow concentration gradient, so only the areas located near the periphery of the prior austenite would transform to martensite, leaving a rather wide portion of the blocky austenite retained upon cryogenic treatment. These result in the transformation ratio of the lath austenite becoming similar to that of the blocky austenite in the IA725 and IA735 specimens.

### Tensile properties

**Figure 4:**
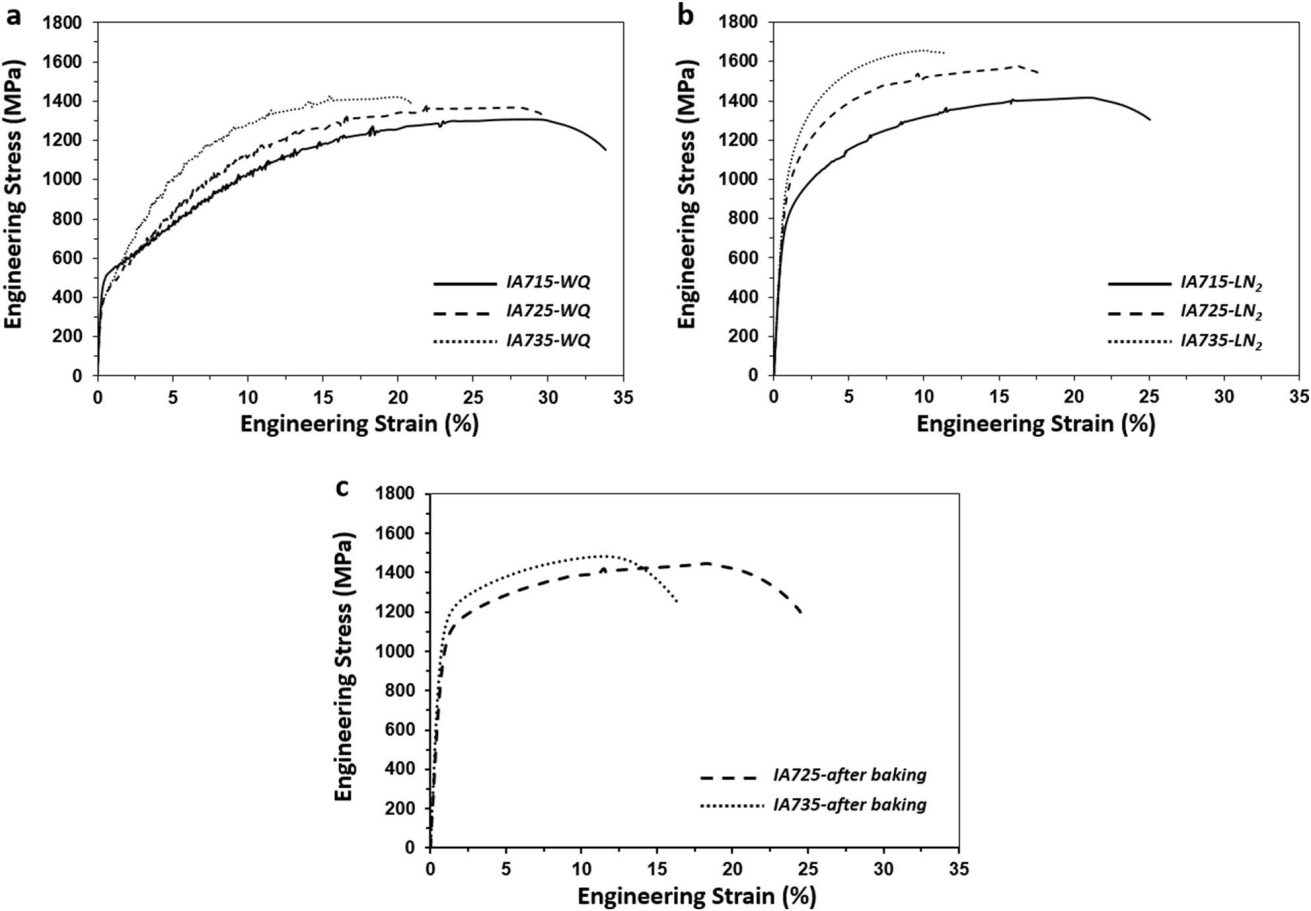
Tensile stress–strain curves of the specimens intercritically annealed (IAed) at different temperatures subjected to water quenching (WQ), cryogenic treatment (LN_2_), and baking treatment. (**a**) WQed, (**b**) cryogenic treated, and (**c**) after baking treatment.

Figure 4 shows the engineering stress–strain curves of the steel in as-IAed condition as well as after cryogenic treatment and their tensile properties are summarized in Table 2. In the water quenched (WQed) condition, yield strength (YS) initially decreases when the IA temperature increases from 715 to 725 °C, while ultimate tensile strength (UTS) increases and elongation decreases. Such behavior is consistent with the results of previous studies on IAed medium Mn TRIP steels^24,28^. With further increasing the IA temperature to 735 °C, there are increases in both YS and UTS, which are mainly due to an increased fraction of martensite with increasing IA temperature. It is noted that all the present specimens subjected to hot rolling followed by IA do not show obvious yield point elongation, which is frequently observed in the cold rolled and IAed medium Mn steels^24,28^. An absence of discontinuous yielding in the hot rolled and IAed specimens is suggested to be mainly due to the simultaneous straining of ferrite and austenite at an early stage of deformation^26^. Discontinuous yielding is known to cause substantial surface roughening^40,41^ and heterogeneous deformation in the coatings that facilitate cracking of the coated layer^41^, and hence continuous yielding behavior of the present steel is more advantageous for automotive applications than discontinuous yielding behavior found in cold rolled and IAed steels.

**Table 2:** Tensile properties and bendability of the specimens intercritically annealed (IAed) at different temperatures subjected to water quenching (WQ), cryogenic treatment (LN_2_) and subsequent baking treatment.

Specimen	IA715		IA725			IA735		
	WQ	LN_2_	WQ	LN_2_	After baking	WQ	LN_2_	After baking
YS (MPa)	518 ± 22	738 ± 3	394 ± 6	871 ± 8	942 ± 3	417 ± 4	974 ± 12	1093 ± 5
UTS (MPa)	1314 ± 4	1433 ± 18	1369 ± 6	1563 ± 16	1447 ± 6	1434 ± 6	1654 ± 15	1513 ± 3
El. (%)	32.5 ± 0.8	24.9 ± 0.7	28.4 ± 0.5	16.6 ± 0.1	23.5 ± 0.2	20.0 ± 0.1	10.4 ± 0.4	15.5 ± 0.4
*R/t* and bending angle (°)	–	–	2.6*	–	82** ± 0.6	5.3*	–	76** ± 0.5

*R/t value; **Bending angle (°)

After cryogenic treatment, there are large increases in both YS and UTS as compared to those in the WQed condition. For example, the IA725-LN_2_ specimen shows YS of 871 MPa (an increase of 477 MPa from the IA725-WQ specimen) and UTS of 1563 MPa (an increase of 194 MPa from the IA725-WQ specimen), with total elongation of ~ 17%. In the case of the IA735-LN_2_ specimen, its YS and UTS values are 974 MPa and 1654 MPa, respectively, with total elongation of ~ 10%. Such large increases in strength of the cryogenic treated specimens compared to that of the WQed specimens are undoubtedly due to the formation of a large amount of martensite induced by cryogenic treatment. The strength of these specimens is quite comparable to that of 22MnB5 HPF steel, while the elongation of the former is higher than that of the latter^6,46^, indicating that it is possible to develop the steel having a better combination of tensile properties than that of HPF steel by cryogenic treatment of IAed steel.

**Figure 5:**
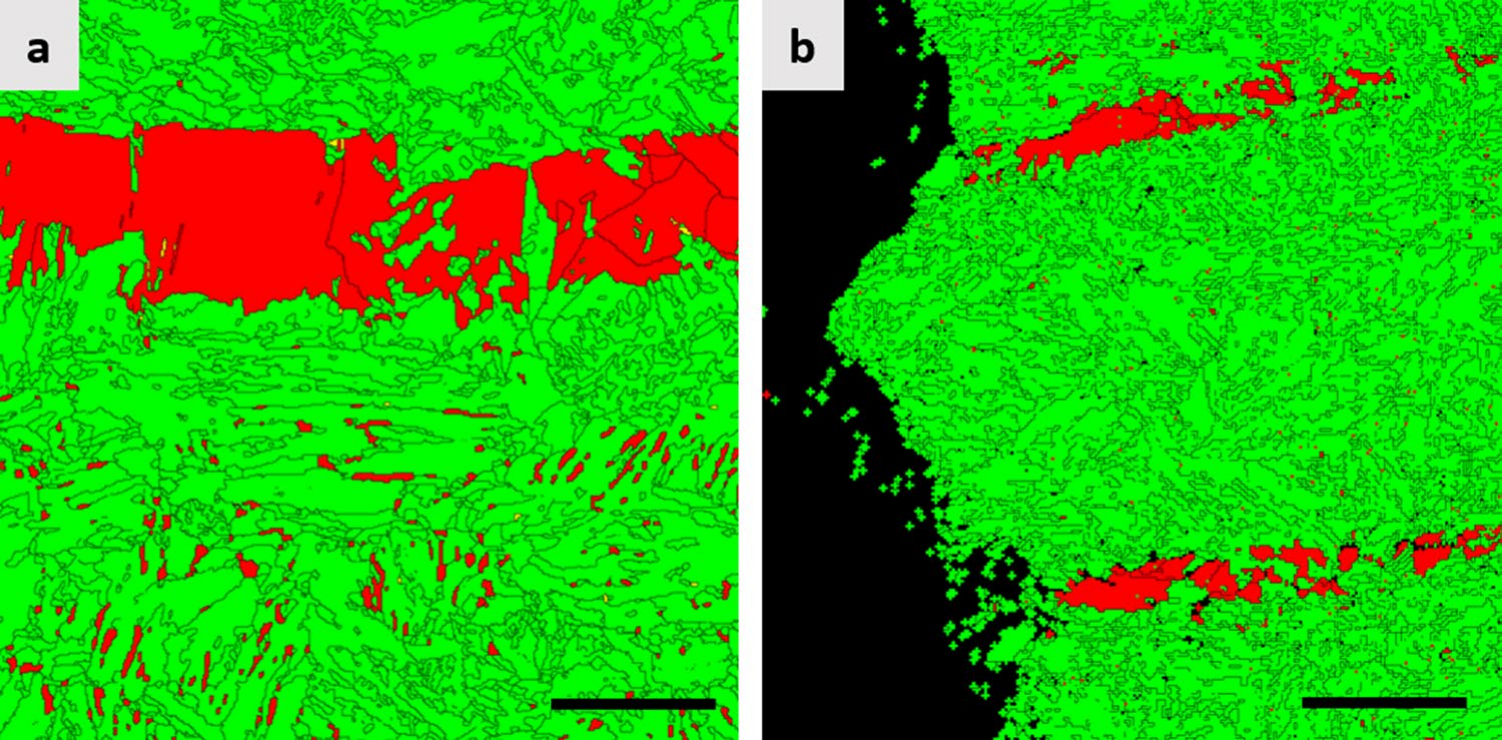
EBSD phase map of the broken tensile and bending specimens. (**a**) tensile specimen intercritically annealed (IAed) at 725 °C followed by cryogenic treatment (tensile direction is horizontal) and (**b**) bending specimen IAed at 725 °C followed by cryogenic treatment and baking treatment (bending axis is perpendicular to the figure). Austenite and bcc phases are marked by red and green colors, respectively. Scale bars in (**a**) and (**b**) correspond to 5 μm and 10 μm, respectively.

Measurement of the fraction of retained austenite in the fractured tensile specimens in cryogenic treated condition by XRD shows that there still exists a substantial amount of austenite (Table 1). As shown in Fig. 5a, while most of the lath austenite transforms to martensite (comparing with Fig. 2f), a rather large amount of the blocky austenite remains untransformed, suggesting that the blocky austenite has a higher mechanical stability than the lath austenite. Such higher mechanical stability of the blocky austenite than that of the lath austenite is mainly due to the former having a much shallower Mn concentration gradient than the latter, as mentioned previously.

### Bendability

As shown above, the present steel shows large elongation values in WQed condition, suggesting its good cold formability. Considering that there is no direct relationship between elongation and formability in AHSS, however, its formability should be evaluated by other methods^47^. In the present study, bending test was performed on the steel in WQed condition and its *R/t* ratio, the minimum recommended inside radius of curvature (*R*) that is necessary to form a 90° bend in a sheet of thickness (*t*) without failure, was measured to evaluate the steel’s formability according to the guidelines of the ISO 7438^38^ and VDA 238-100^39^, which are the standards in the industry. Bending tests can also be used to evaluate the steel’s crashworthiness, as demonstrated by the works of Till et al.^48^ and Manuel et al.^49^ showing a clear relationship between full-scale crash test results and the bendability, i.e., bending angle, of AHSS. Hence, the bending test was also conducted on the steel in cryogenic treated condition and its bending angle was measured to evaluate the steel’s crashworthiness. It is to be noted that the bending test was conducted with the worst specimen configuration such that the bending axis is in the longitudinal direction, which normally gives worse properties than the one in the transverse direction^50,51^. Only the IA725 and IA735 specimens were subjected to the bending test since the IA715-LN_2_ specimen has much lower strength than the HPF steels.

Bending tests of the specimens in WQed conditions show that the IA725-WQ and IA735-WQ specimens have *R/t* ratios of 2.6 and 5.3, respectively. *R/t* ratio is typically used for the evaluation of formability of various AHSS^34,52^ and the lower *R/t* value indicates the better formability. *R/t* ratio of the IA725-WQ specimen is lower than those of the cold-formable 1400 MPa grade martensitic steel (*R/t* = 4) and even the cold-formable 1000 MPa grade dual phase steels (*R/t* = 3)^34,52^, indicating its good formability at room temperature. On the other hand, the IA735-WQ specimen would have a difficulty in cold forming.

In the case of the cryogenic treated specimens, bending test as well as additional tensile test were conducted after baking the specimens for 170 °C for 20 min to simulate the heating that occurs in the body paint line^35,36,53^, whose reason for such heat treatment is explained as follows. Once the steel sheet is stamped to a net shape component, it would be subjected to painting followed by curing, which is known as the paint baking cycle. It is usually conducted at 170 °C for 20 min, which would change the properties of the components. It has been reported that the YS of the HPF steels increases by about 100 MPa by bake hardening^34–36^. Measurement of the bending angles for the cryogenic treated specimens after simulated baking process shows that the IA725-LN_2_ and IA735-LN_2_ specimens have bending angles of 82° and 76°, respectively, which are much larger than that (55°) of the 22MnB5 HPF steel^52^, demonstrating the present steel’s excellent crashworthiness. Observation of the fractured IA725-LN_2_ bending specimen subjected to baking (Fig. 5b) shows that the blocky austenite is still retained without being transformed to martensite, similar to the result of tensile test performed on the IA725-LN_2_ specimen. Such high mechanical stability of austenite in the baked IA725-LN_2_ specimen is presumably due to an enhancement of stability of austenite by baking^54^. It is noted that even the IA735-LN_2_ specimen shows good crashworthiness after baking treatment, although its room temperature formability, the *R/t* ratio, is lower than that of the cold-formable martensitic steels having a similar strength level.

**Figure 6:**
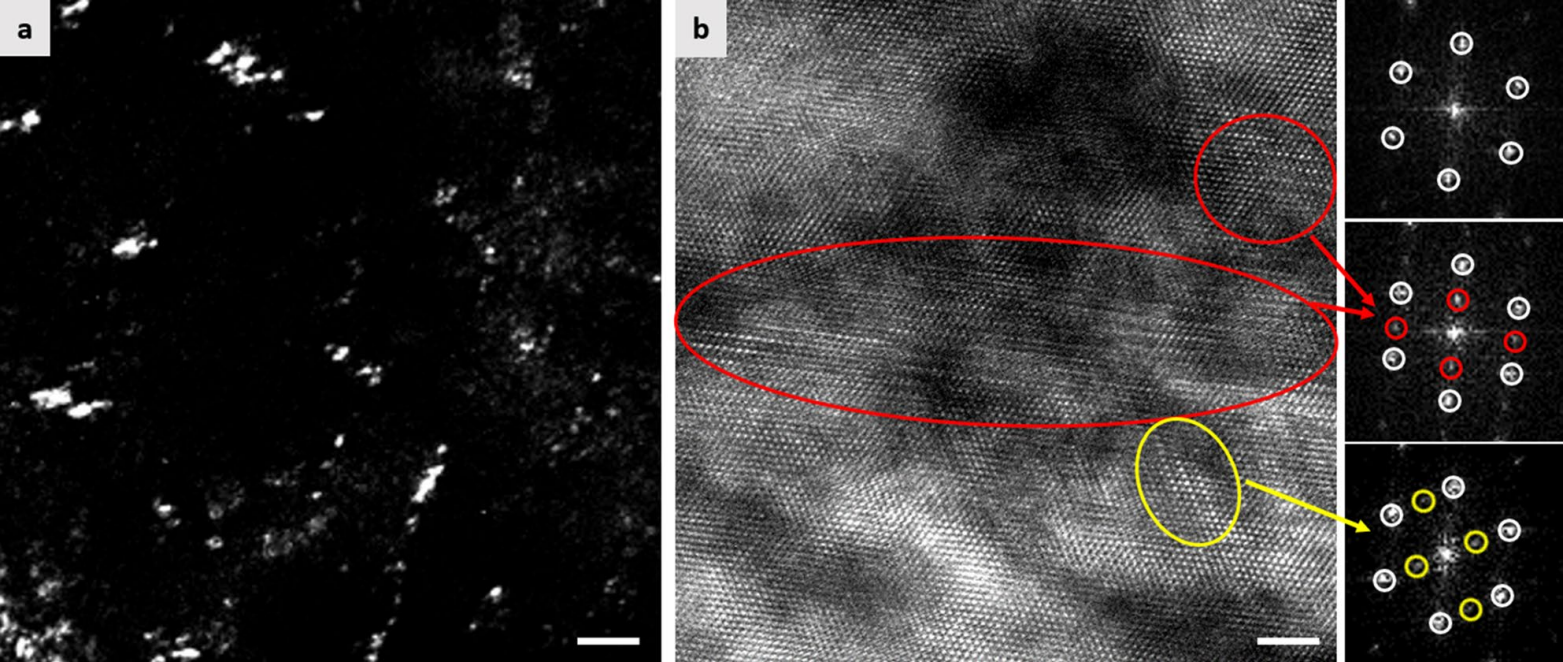
TEM micrographs showing the precipitation of ε-carbide particles in the specimen intercritically annealed at 725 °C followed by cryogenic treatment and baking treatment. (**a**) Dark field image of ε-carbide particles. (**b**) High resolution image of martensite and ε-carbide particles and their corresponding fast Fourier transform patterns. White circle: diffraction spots of martensite in [111] zone axis. Red circle: diffraction spots of the first variant of ε-carbide in [$2\overline{11 }0$] zone axis. Yellow circle: diffraction spots of the second variant of ε-carbide in [$2\overline{11 }0$] zone axis. Scale bars in (**a**) and (**b**) correspond to 20 nm and 2 nm, respectively.

As mentioned previously, it has been reported that there is a change in tensile properties of the HPF steels by baking, which might also happen in the present type of steel. Therefore, tensile properties of the IA725-LN_2_ and IA735-LN_2_ specimens were evaluated after baking at 170 °C for 20 min. It shows that there is indeed a change in tensile properties as shown in Table 2. After baking, both IA725-LN_2_ and IA735-LN_2_ specimens show increases in YS (by 71 MPa and 143 MPa for the IA725-LN_2_ and IA735-LN_2_ specimens, respectively) and elongation (by 6.9% and 4.1% for the IA725-LN_2_ and IA735-LN_2_ specimens, respectively). Previous studies showed that there is no apparent changes in the fraction of austenite in medium Mn TRIP steels subjected to baking treatments^29,30^. TEM analysis of the baked IA725-LN_2_ specimen, however, shows that there is a precipitation of ε-carbide in martensite (Fig. 6), increasing the strength of martensite. In addition, partitioning of C atoms from ferrite and martensite to austenite during baking increases the strength of austenite^55^. Both of these changes in the microstructure would lead to an increase in YS after baking. In addition, increased mechanical stability of austenite by C partitioning^54,55^ would lead to an increase in elongation after baking, in contrast to the 22MnB5 HPF steel showing a decrease in elongation after baking^36,53^.

In summary, our alloy and processing design concepts enable the development of the steel having an excellent combination of strength and formability, which are mutually contradicting properties. In addition, the developed steel shows excellent crashworthiness, which is crucial for the safety of passengers in automobiles. Furthermore, the developed steel has a composition that is quite similar to those of other commercial TRIP steels and its processing route does not involve high temperature forming causing harmful interactions between coating and substrates so that the developed steel would be expected to gain acceptance by automotive manufactures.

## Data Availability

All data supporting the findings of this study are available from the corresponding author on request.
